# Relationship between food group-specific intake and depression among local government employees in Japan

**DOI:** 10.1186/s40795-024-00830-4

**Published:** 2024-01-30

**Authors:** Makiko Kitabayashi, Shoko Umetsu, Miho Suzuki, Tsuneo Konta

**Affiliations:** 1 Department of Health and Nutrition, Yamagata Prefectural Yonezawa University of Nutrition Sciences, Yonezawa, Yamagata Japan; 2 Department of Public Health and Hygiene, Yamagata University Graduate School of Medical Sciences, Yamagata, Yamagata Japan

**Keywords:** Depression, Vegetables, Eggs, Odds ratio, Center for Epidemiologic Studies Depression scale (CES-D)

## Abstract

**Background:**

We aimed to examine the relationship between food group-specific intake and depression among workers in Japan.

**Methods:**

A questionnaire survey was administered to 568 workers in 2020; 503 workers responded and 423 were included in the study. Information on sex, age, body mass index, overtime hours, sleep duration, marital status, employment position, exercise habits, smoking status, incidence of depression, and intake of energy, proteins, lipids, carbohydrates, alcohol, and specific food groups were collected. The Center for Epidemiologic Studies Depression Scale was used to assess the presence and severity of depression. Food group-specific intake was adjusted for energy intake using the residual method and classified into low, moderate, and high by sex. Logistic regression was used to examine the odds ratios (ORs) and trends according to sex, with the presence/absence of depression as the dependent variable and food group-specific intake as the independent variable.

**Results:**

Men in the eggs low-intake, and women in the other vegetables low- and moderate-intake and eggs moderate-intake groups had significantly higher adjusted ORs (aORs) for depression. Additionally, a dose-response relationship was observed, where the OR for depression was significantly higher in men when the intake of eggs was low (p for trend = 0.024) and in women when the intakes of other vegetables (p for trend = 0.011) and eggs (p for trend = 0.032) were low.

**Conclusions:**

The intake of eggs in men and eggs and vegetables in women may be related to depression.

## Background

The Japanese Ministry of Health, Labour and Welfare mentioned the important mental and physical health issues among employees. There is an upward tendency in the rate of positive findings at regular health checkups; approximately one in two men and one in five women are strongly suspected of having or being at risk of metabolic syndrome, which triggers heart disease and cerebrovascular disease. Furthermore, the proportion of employees who experience excessive work-related anxiety and stress is relatively high [1]. The Ministry also indicated the importance of addressing the mental and physical health issues of employees at an early stage by implementing preventative measures [1]. According to the 2020 Ministry of Health, Labour and Welfare Occupational Safety and Health Survey [2], 7.8% of companies had employees who have taken a leave of absence for 1 month or longer due to poor mental health from November 2019 to October 2020—an increase from 6.7% in the previous year. Furui et al. [3] reported a relationship between employee health risks and loss of labor productivity. The promotion of mental health measures for employees is crucial for improving labor productivity.

Suicide is one of the mental health issues among workers. Health problems account for most of the cases of suicide, and depression is frequently present in the majority of people who die by suicide [4]. Therefore, the prevention of depression is considered an important mental health-related measure for employees. Diet, sleep, and exercise have been associated with the risk of depression. Individuals with low levels of physical activity have a high risk of developing depression [5], while those who are more physically active show a lower prevalence and incidence of depression [6]. With regard to sleep, studies that targeted the older population revealed a significant relationship between sleep disorders and depression in both men and women, and a relationship between sleep disorders and depression in women alone when analyzed by sex [7].

In terms of food, iron [8–10], zinc [10–12], magnesium [10, 13], n-3 polyunsaturated fatty acids [14–17], vitamin D [18], folic acid [17, 19], and vitamin B_12_ [17, 19] have been associated with depression and depressive symptoms. Relevant studies also revealed a relationship between iron deficiency and the onset of depression, between zinc deficiency and the risk of depression, and between low magnesium intake and depressive symptoms. Furthermore, zinc is an effective supplement for treating depression [11], and oral administration of folic acid and vitamin B_12_ has also been recommended [19]. There is accumulating evidence supporting the relationship between nutrients and depression; however, studies related to food group-specific intake are limited. To prevent depression, it is important to identify the types of food that are consumed daily and to examine the different food groups and dietary patterns. Many studies have shown that a higher intake of vegetables and fruits lowers the risk of depression [20–27]. However, previous studies that reported on food group-specific intake other than that of vegetables or fruits only evaluated the intake of seafood [15, 16, 25, 28] and dairy [25, 29]. A study in rats revealed that whole eggs may prevent and alleviate depression [30]. Additionally, a study among Iranian participants showed that mental disorders such as depression and anxiety were less likely to occur in individuals with a higher intake of eggs, fruits, dairy, yogurt, and vegetables [31]. However, to the best of our knowledge, no studies have examined the relationship between food group-specific intakes and depression among Japanese employees.

Therefore, this cross-sectional study aimed to clarify the relationship between food group-specific intake and depression among employees according to sex.

## Methods

### Survey period and participants

We conducted a questionnaire-based survey among local government employees from October to November 2020.The target population comprised all employees of the Yonezawa City hall in Yamagata. The purpose of the survey was explained in writing to all 568 employees of the city hall of Yonezawa City, and their cooperation was requested. Survey questionnaires were distributed and collected at a city hall in cooperation with the General Affairs Department. Envelopes containing the questionnaires were sealed to prevent the leakage of personal information. In total, 503 individuals who provided written informed consent and responded to the survey were included in the study (88.6% consent rate). Participants with significant data loss were requested to re-enter their data.

**Fig. 1:**
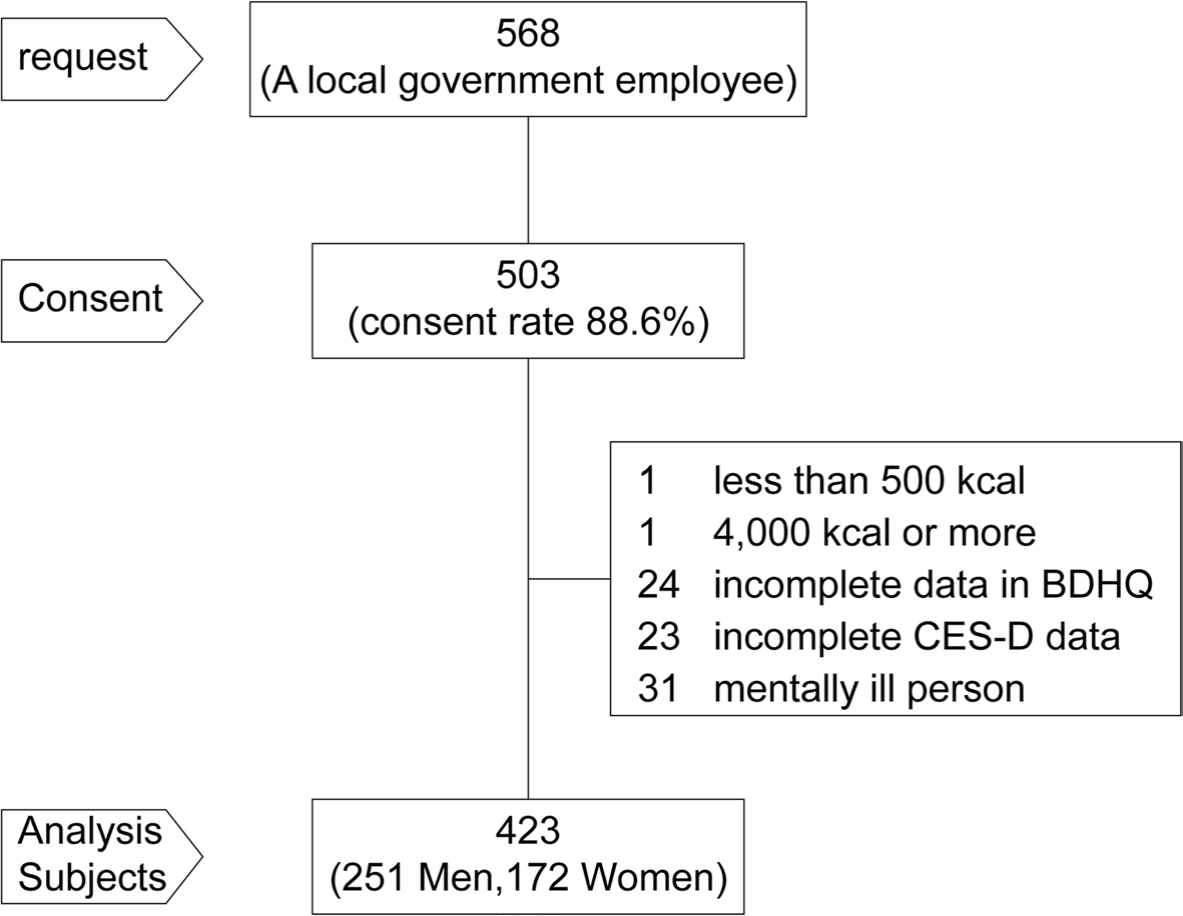
Flow chart for the inclusion of participants in the study. Of 503 that responded, the data collected from 423 participants were analyzed. Of those, data from 80 participants were excluded: two did not meet the required energy intake, 24 had incomplete dietary survey data, 23 had incomplete the Center for Epidemiologic Studies Depression scale (CES-D) data, and 31 had mental illnesses such as depression. The CES-D scale is a short self-report scale to assess depressive symptoms in the general population

Studies conducted among adults in Japan often exclude individuals with intakes <600 kcal or >4,000 kcal, as this is considered insufficient or excessive daily energy intake, respectively [32, 33]. We followed the same practice. Of the 503 participants who responded, 80 were excluded—two did not meet the required energy intake, 24 had incomplete dietary survey data (there was no data on nutrients), 23 had incomplete CES-D data (there was no depression assessment data), and 31 had mental illnesses such as depression. Hence, only the data of 423 individuals (251 men and 172 women; analysis rate, 84.1%) were analyzed. The presence or absence of mental illness, such as depression, was determined by inquiring about the history of depression and other mental illnesses using a lifestyle habit questionnaire. The recruitment of participants is shown in Fig. 1.

### Survey items

A self-administered questionnaire on lifestyle habits together with a brief self-administered diet history questionnaire (BDHQ), developed by Sasaki et al. [34–37], was used for the survey. The BDHQ is a relatively simple, individualized survey of the amount of nutrients consumed in the habitual diet of people living in Japan. This questionnaire allows for the quantitative and detailed examination of nutrient and food intake status. By respondents reporting the foods consumed over the previous month, their intake of energy, water, and approximately 100 nutrients and 67 food items can be calculated. Responses to the 67 foods from a typical Japanese diet were recorded based on the following categories: “at least twice a day,” “once a day,” “4–6 times a week,” “2–3 times a week,” “once a week,” “less than once a week,” or “never ate.”

### Participant attributes and lifestyle habits

The categorical variables included sex, marital status (married or unmarried), employment position (manager or general worker), exercise habits (presence or absence of regular exercise), and smoking habits (current smoker, previous smoker, or non-smoker). The continuous variables included age, height (cm), weight (kg), daily hours of overtime worked, and daily hours of sleep. Body mass index (BMI) (kg/m^2^) was calculated using the participants’ height and weight.

### Depression

Depression was assessed using the Japanese version of the Center for Epidemiologic Studies Depression scale (CES-D) [38]. The CES-D comprises a total of 20 items: 16 negative items, such as depressed mood, interpersonal relationships, and physical symptoms, and 4 positive items, such as life satisfaction and life enjoyment [39, 40]. For each item, the frequency of experience in the previous week was divided into four categories: “none” (0 points), “1–2 days” (1 point), “3–4 days” (2 points), and “5 days or more” (3 points). The total score was defined as the CES-D score. Participants were dichotomized into those with a score ≥16 points (classified as having depression) and those with a score <16 points [38, 41]. In a systematic review and meta-analysis of 28 studies, at a cutoff of 16 points, the sensitivity was 0.87 (95% confidence interval [CI]: 0.82–0.92); specificity, 0.70 (95% CI: 0.65–0.75); and diagnostic odds ratio (DOR), 16.2 (95% CI: 10.49–25.10) [42]. The DOR summarizes the diagnostic accuracy of a test by providing a single number indicating how many times higher the odds are of obtaining a positive test result in a diseased person as compared to a non-diseased person.

### Dietary intake survey

At the DHQ Support Center [43], BDHQ responses were analyzed and tabulated to calculate the energy (kcal), protein (g), fat (g), carbohydrate (g), and alcohol (g) intakes. Each of the protein, fat, and carbohydrate intakes was divided by the total energy intake to calculate the energy ratio (%E). Additionally, data on the intake (g) of 15 food groups (cereals, potatoes, sugars, legumes, green and yellow vegetables, other vegetables, total vegetables, fruits, seafood, meat, eggs, dairy, fats and oils, sweets, and preferred beverages) were collected. Since nutrient intake is often positively correlated with total energy intake, energy adjustments were made using the residual method to remove the influence of total energy intake on nutrient intake. The residual method corrects for the effect of differences in energy intakes by using a linear regression equation of total energy intake on the nutrient intake of interest, and adding the amount of the nutrient of interest when the average energy intake is taken as the residual difference between the predicted and measured values of interest obtained from this linear regression equation [44]. The food group-specific intake values were classified into tertiles (low, moderate, and high) according to sex.

### Statistical analyses

IBM SPSS Statistics for Windows version 26.0 (IBM, Armonk, NY, USA) was used for statistical analysis. A two-tailed test was conducted, with a statistical significance level of 5%. The Kolmogorov–Smirnov test was used to test for normality. For group differences by sex, as well as group differences by the presence or absence of depression and sex, the t-test was used for continuous variables with a normal distribution, while the non-parametric Mann–Whitney U test was used for those with a non-normal distribution. For categorical variables, the chi-square test was used.

Logistic regression analysis was used to set Model I. The presence or absence of depression was assigned as the dependent variable (absence used as the reference group) and the three intake level groups (high, moderate, and low) subdivided into four food groups (potatoes, other vegetables, meat, and eggs) as the independent variables for each food group; no moderator variables were used. The odds ratios (ORs) and 95% CIs for each sex, with the high-intake group as the reference group, as well as the trends were determined, and the *p* for trend was calculated for each of the four food groups. We assessed for multicollinearity issues, and confirmed that there was no strong correlation between the four food groups; subsequently, the four groups were simultaneously entered into the model. For Model II, age, sleep duration, exercise habits, alcohol intake, and smoking, which may be related to food group-specific intake or depression, were added to Model I as moderator variables. Similar to Model I, the ORs and 95% CIs for each sex and trends were determined, and the *p* for trend was calculated. The trends were measured by obtaining the regression coefficient using a logistic regression analysis; linear scores (the median of the tertiles) were assigned to the three food group-specific intake groups. Data are expressed as the mean ± standard deviation when normally distributed and as the median (25^th^–75^th^ percentile) when non-normally distributed. Categorical variables are expressed as percentages.

## Results

### Participant characteristics

Table 1 shows the characteristics of the participants according to sex. The items that did not differ between men and women were overtime hours, sleep duration, and marital status. Age, BMI, energy intake, carbohydrate intake (%E), alcohol intake, and the percentage of managers, individuals who exercised regularly, and current smokers were significantly higher among men; meanwhile, the CES-D score and intake of proteins (%E) and fats (%E) were significantly higher among women. The mean overall CES-D score was 13.0 points (men: 12.1 points, women: 14.3 points). Overall, the percentage of participants with depression (CES-D score ≥16 points) was 28.4% (*n*=120), of whom 24.3% (*n*=61) were men and 34.3% (*n*=59) were women. The CES-D scores were significantly higher among women (*p* = 0.028). Table 2 shows the characteristics of the participants, divided by sex, according to the presence or absence of depression. A significant difference was found in the sleep duration in both men and women depending on the presence or absence of depression. In men, the mean sleep durations were 6.6 ± 1.0 hours for those without depression and 6.5 ± 1.2 hours for those with depression; men with depression slept significantly fewer hours (*p* < 0.001). Similarly, women without depression slept 6.5 ± 1.1 hours, while those with depression slept 6.2 ± 1.1 hours; women with depression slept significantly fewer hours (*p* < 0.001). In men, 41.7% and 56.1% of those with and without depression, respectively, exercised regularly; those without depression tended to perform exercise more frequently (*p* = 0.055). In women, no significant difference was observed in the frequency of exercise between those with depression and those without depression.

**Table 1:** Participant characteristics

Characteristic			Total		Men		Women		*p*-value
			(*n*=423)		(*n*=251)		(*n*=172)		
Age	Mean ± SD, years		42.1 ± 10.8		43.1 ± 10.5		40.7 ± 11.2		0.026
Body mass index	Mean ± SD, kg/m^2^		22.9 ± 3.6		23.8 ± 3.6		21.5 ± 3.1		<0.001
Overtime hours	Mean (25^th^–75^th^ percentile), hours/month		5.0 (0.0–20.0)		5.0 (0.0–20.0)		5.0 (0.0–15.0)		0.764
Sleep duration	Mean ± SD, hours/day		6.5 ± 1.1		6.6 ± 1.0		6.4 ± 1.1		0.055
CES-D score	Mean ± SD		13.0 ± 8.3		12.1 ± 7.5		14.3 ± 9.1		0.010
Energy intake	Mean ± SD, kcal		1,848 ± 557		1,961 ± 573		1,681 ± 487		<0.001
Protein intake	Mean ± SD, %E		15.1 ± 2.6		14.6 ± 2.6		15.7 ± 2.5		<0.001
Fat intake	Mean ± SD, %E		26.1 ± 5.6		25.1 ± 5.8		27.6 ± 4.9		<0.001
Carbohydrate intake	Mean ± SD, %E		58.8 ± 7.5		60.2 ± 7.8		56.7 ± 6.6		<0.001
Alcohol intake	Median (25^th^–75^th^ percentile), g		2.5 (0.0–17.6)		7.3 (0.8–29.4)		0.2 (0.0–3.5)		<0.001
Depression	Present	n (%)	120	(28.4)	61	(24.3)	59	(34.3)	0.028
	Absent	n (%)	303	(71.6)	190	(75.7)	113	(65.7)	
Marital status	Married	n (%)	295	(69.7)	179	(71.3)	116	(67.4)	0.451
	Unmarried	n (%)	128	(30.3)	72	(28.7)	56	(32.6)	
Employment position	Manager	n (%)	39	(9.5)	36	(14.8)	3	(1.8)	<0.001
	General staff	n (%)	373	(90.5)	207	(85.2)	166	(98.2)	
Regular exercise	Present	n (%)	185	(44.7)	130	(52.6)	55	(32.9)	<0.001
	Absent	n (%)	229	(55.3)	117	(47.4)	112	(67.1)	
Smoking status	Non-smoker	n (%)	290	(69.0)	125	(50.4)	165	(95.9)	<0.001
	Previous smoker	n (%)	74	(17.6)	71	(28.6)	3	(1.7)	
	Current smoker	n (%)	56	(13.2)	52	(21.0)	4	(2.3)	

*CES-D*: Center for Epidemiologic Studies Depression

Continuous variables: normal distribution, t-test; non-normal distribution, Mann–Whitney U test.

Categorical variables: chi-square test

Significance level < 0.05 (two-sided test)

**Table 2:** Participant characteristics according to the presence or absence of depression

			Men			Women		
			Depression absent	Depression present	*p-value*	Depression absent	Depression present	*p-value*
			*n*=190	*n*=61		*n*=113	*n*=59	
CES-D score	mean ± SD, n		8.7 ± 4.0	22.6 ± 6.1		9.0 ± 4.0	24.3 ± 7.5	
Age^a^	mean ± SD, years		43.3 ± 10.6	42.5 ± 10.3	0.993	40.6 ± 11.0	41.0 ± 11.6	0.909
Body mass index^a^	mean ± SD, kg/m^2^		23.8 ± 3.6	23.9 ± 3.6	0.885	21.5 ± 3.2	21.6 ± 3.0	0.763
Overtime hours^b^	median (25th–75th percentile), hours/month		5.0 (0.0–20.0)	5.0 (1.0–20.0)	0.676	5.0 (0.0–17.0)	2.0 (0.0–15.0)	0.096
Sleep duration^a^	mean ± SD, hours/day		6.6 ± 1.0	6.5 ± 1.2	<0.001	6.5 ± 1.1	6.2 ± 1.1	<0.001
Energy intake^a^	mean ± SD, kcal		1,944 ± 552	2,016 ± 633	0.164	1,713 ± 490	1,620 ± 481	0.444
Protein intake^a^	mean ± SD, %E		14.8 ± 2.6	14.3 ± 2.8	0.315	15.8 ± 2.5	15.4 ± 2.4	0.143
Fat intake^a^	mean ± SD, %E		25.3 ± 5.7	24.6 ± 6.1	0.175	27.8 ± 4.8	27.3 ± 5.0	0.301
Carbohydrate intake^a^		mean ± SD, %E	60.0 ± 7.7	61.1 ± 8.2	0.279	56.3 ± 6.4	57.4 ± 6.8	0.570
Alcohol intake	Median (25th–75th percentile), g		8.3 (1.2–30.8)	4.5 (0.6–24.2)	0.151	0.5 (0.0–4.5)	0.2 (0.0–3.1)	0.398
Marital status^c^	Married	n (%)	139 (73.2)	40 (65.6)	0.259	76 (67.3)	40 (67.8)	1.000
	Not married	n (%)	51 (26.8)	21 (34.4)		37 (32.7)	19 (32.2)	
Employment position^c^	Manager	n (%)	30 (16.4)	6 (10.0)	0.296	2 (1.8)	1 (1.7)	1.000
	General staff	n (%)	153 (83.6)	54 (90.0)		108 (98.2)	58 (98.3)	
Regular exercise^c^	Present	n (%)	105 (56.1)	26 (41.7)	0.055	40 (36.7)	15 (25.9)	0.121
	Absent	n (%)	81 (43.9)	35 (58.3)		69 (63.3)	43 (74.1)	
Smoking status^c,d^	Non-smoker	n (%)	91 (49.2)	33 (55.0)	0.490	108 (95.6)	57 (96.6)	0.923
	Previous smoker	n (%)	55 (29.7)	14 (23.3)		2 (1.8)	1 (1.7)	
	Current smoker	n (%)	39 (21.1)	13 (21.7)		3 (2.7)	1 (1.7)	

*CES-D* Center for Epidemiologic Studies Depression

^a^t-test

^b^Mann–Whitney U test

^c^chi-square test

^d^Fisher's exact test, Significance level < 0.05 (two-sided test)

Depression absent (CES-D score <16), Depression present (CES-D score ≥16)

### Food group-specific intake according to the presence or absence of depression

Table 3 shows the results of the comparison between the intakes of 15 food groups adjusted for energy intake according to the presence or absence of depression and sex. The intakes of potatoes (*p* = 0.036), other vegetables (*p* = 0.037), total vegetables (*p* = 0.040), meat (*p* = 0.044), and eggs (*p* = 0.015) were significantly lower in men with depression than in men without depression. In women, no significant relationship was found between the intake of specific food groups and the presence or absence of depression.

**Table 3:** Food group-specific intake according to the presence or absence of depression

		Men			Women		
		CES-D <16	CES-D ≥16	*p*-value	CES-D <16	CES-D ≥16	*p*-value
		*n*=190 (75.7%)	*n*=61 (24.3%)		*n*=113 (65.7%)	*n*=59 (34.3%)	
Cereals^a^	(g)	416.1 ± 137.8	433.5 ± 169.2	0.205	384.7 ± 112.4	397.4 ± 114.6	0.453
Potatoes^b^	(g)	40.7 (24.0–61.2)	32.2 (13.4–58.6)	0.036	51.4 (30.5–83.7)	49.8 (26.4–66.8)	0.661
Sugars^b^	(g)	2.9 (1.6–4.6)	2.6 (1.8–4.2)	0.593	3.7 (2.4–4.9)	3.5 (2.2–5.3)	0.924
Legumes^b^	(g)	71.5 (46.3–99.0)	58.1 (32.1–97.0)	0.122	69.0 (47.5–97.7)	65.5 (44.6–101.4)	0.950
Green and yellow vegetables^b^	(g)	75.9 (53.6–113.1)	65.9 (41.1–102.4)	0.146	97.6 (66.5–143.9)	84.7 (65.7–119.6)	0.218
Other vegetables^b^	(g)	125.3 (90.4–167.5)	103.3 (75.4–150.4)	0.037	154.6 (123.5–209.7)	138.6 (116.5–169.8)	0.067
Total vegetables^b^	(g)	206.3 (150.1–282.0)	161.7 (120.4–263.6)	0.040	245.3 (190.5–341.2)	223.5 (190.5–277.6)	0.123
Fruits^b^	(g)	60.4 (28.0–113.7)	62.2 (29.6–127.3)	0.802	85.5 (53.0–130.8)	88.5 (55.0–126.0)	0.828
Seafood^b^	(g)	64.8 (47.4–87.9)	71.0 (38.6–90.2)	0.881	71.8 (52.8–97.4)	68.2 (48.7–95.6)	0.655
Meat^b^	(g)	73.5 (51.4–100.4)	67.3 (44.3–86.0)	0.044	83.4 (64.6–102.5)	76.5 (60.8–94.7)	0.291
Eggs^b^	(g)	35.3 (24.8–56.4)	25.1 (16.1–50.3)	0.015	40.6 (23.6–53.7)	33.5 (24.8–47.2)	0.497
Dairy^b^	(g)	96.0 (27.3–180.6)	65.1 (21.8–136.7)	0.255	127.0 (47.6–162.9)	120.6 (66.3–165.9)	0.838
Fats and oils^b^	(g)	11 (7.7–13.5)	10.4 (6.8–13.8)	0.479	10.5 (8.1–13.2)	11.0 (8.7–14.0)	0.501
Sweets^b^	(g)	30.9 (12.7–50.0)	36.1 (16.6–58.9)	0.116	32.3 (18.1–52.0)	39.5 (14.8–59.2)	0.723
Preferred beverages^b^	(g)	644.2 (443.9–980.8)	681.0 (434.2–875.8)	0.558	496.3 (362.0–693.4)	448.3 (267.9–668.8)	0.403

Food group-specific intake (g) is adjusted for energy intake using the residual method.

^a^Mean ± standard deviation, t-test, significance level of <0.05 (two-tailed test)

^b^Median (25^th^–75^th^ percentile), Mann–Whitney U test, significance level of <0.05 (two-tailed test)

### Relationship between food group-specific intake and depression

In men, significant differences were found in the intake of potatoes, other vegetables, total vegetables, meat, and eggs between those with and without depression (Table 3). Therefore, ORs were calculated with the intake of these food groups as the independent variables and the presence of depression as the dependent variable. Since total vegetables include other vegetables, the analysis was conducted for only four of the food groups (potatoes, other vegetables, meat, and eggs), and not for total vegetables. Table 4 shows the number, median, minimum, and maximum values for low, moderate, and high intake of the four food groups adjusted for energy intake by sex. The relationship between food group-specific intake and depression in men and women is shown in Tables 5 and 6, respectively. In Model I (not adjusted for background factors), significantly higher ORs (95% CIs) for depression were found in the following groups compared with the high-intake group: in men, the eggs low-intake group (2.29 [1.13–4.63]) and in women, the other vegetables low-intake group (2.68 [1.11–7.36]), other vegetables moderate-intake group (2.66 [1.12–6.28]), and eggs moderate-intake group (2.52 [1.08–5.90]). A dose-response relationship was observed; that is, the OR for depression was significantly higher in men with a low intake of eggs (*p* for trend = 0.038) and in women with a low intake of other vegetables (*p* for trend = 0.013).

**Table 4.** Food group-specific intake values by tertiles (low, moderate, and high)

		Men (*n*=251)				Women (*n*=172)			
		n	Median (g)	Minimum (g)	Maximum (g)	n	Median (g)	Minimum (g)	Maximum (g)
Potatoes	Low-intake group	83	13.4	-44.4	27.8	57	24.8	4.2	35.4
	Moderate-intake group	84	38.3	27.9	54.9	58	51.1	36.8	59.9
	High-intake group	84	67.4	55.2	156.4	57	92.5	60.3	258.4
Other vegetables	Low-intake group	83	71.9	-12.7	98.4	57	103.5	2.9	127.5
	Moderate-intake group	84	118.2	98.9	147.0	58	143.7	128.2	177.9
	High-intake group	84	201.4	148.0	604.4	57	224.6	179.7	591.9
Meat	Low-intake group	83	42.2	-18.1	56.1	57	54.3	0.3	67.3
	Moderate-intake group	84	71.6	56.4	86.3	58	77.0	67.3	92.0
	High-intake group	84	108.8	86.3	350.0	57	106.0	92.3	381.3
Eggs	Low-intake group	83	16.8	-21.7	25.7	57	20.3	-4.4	27.9
	Moderate-intake group	85	33.7	25.8	47.9	58	35.3	28.0	48.4
	High-intake group	83	64.0	48.5	156.4	57	58.5	48.6	130.4

Food group-specific intake (g) is adjusted for energy intake using the residual method; food group-specific intake is classified into three groups (low-intake group, moderate-intake group, high-intake group)

**Table 5:** Relationship between food group-specific intake and depression (men)

Food group		Men (*n*=251)							
		Number of men with depression (%)		Model I^a^			Model II^b^		
				Odds ratio (95% confidence interval)		*p* for trend	Odds ratio (95% confidence interval)		*p* for trend
Potatoes	Low-intake group	25	(30.1)	1.25	(0.58–2.72)	0.390	1.42	(0.63–3.23)	0.274
	Moderate-intake group	19	(22.6)	0.91	(0.41–2.00)		0.94	(0.41–2.15)	
	High-intake group	17	(20.2)	1.00			1.00		
Other vegetables	Low-intake group	28	(33.7)	1.75	(0.81–3.77)	0.174	1.51	(0.67–3.42)	0.284
	Moderate-intake group	17	(20.2)	1.08	(0.49–2.39)		1.02	(0.44–2.35)	
	High-intake group	16	(19.0)	1.00			1.00		
Meat	Low-intake group	25	(30.1)	1.54	(0.71–3.32)	0.197	1.67	(0.75–3.72)	0.143
	Moderate-intake group	21	(20.2)	1.50	(0.68–3.31)		1.70	(0.74–3.88)	
	High-intake group	15	(25.0)	1.00			1.00		
Eggs	Low-intake group	32	(38.6)	2.29	(1.13–4.63)	0.038	2.59	(1.21–5.54)	0.024
	Moderate-intake group	12	(14.3)	0.68	(0.30–1.55)		0.76	(0.32–1.77)	
	High-intake group	17	(20.5)	1.00			1.00		

Food group-specific intake (g) is adjusted for energy intake using the residual method; food group-specific intake is classified into three groups (low-intake group, moderate-intake group, high-intake group)

^a^Model I: not adjusted for background factors

^b^Model II: adjusted for age, sleep hours, regular exercise, alcohol consumption, smoking

Multiple logistic regression analysis; Dependent variable: presence or absence of depression; Significance level <0.05 (two-sided test)

**Table 6:** Relationship between food group-specific intake and depression (Women)

Food group		Women (*n*=172)							
		Number of women with depression (%)		Model I^a^			Model II^b^		
				Odds ratio (95% confidence interval)		*p* for trend	Odds ratio (95% confidence interval)		*p* for trend
Potatoes	Low-intake group	25	(36.8)	0.68	(0.28–1.66)	0.469	0.68	(0.28–1.66)	0.300
	Moderate-intake group	19	(31.0)	0.60	(0.25–1.46)		0.60	(0.25–1.46)	
	High-intake group	17	(35.1)	1.00			1.00		
Other vegetables	Low-intake group	28	(40.4)	2.86	(1.11–7.36)	0.013	2.86	(1.11–7.36)	0.011
	Moderate-intake group	17	(41.4)	2.72	(1.09–6.82)		2.72	(1.09–6.82)	
	High-intake group	16	(21.2)	1.00			1.00		
Meat	Low-intake group	25	(35.1)	1.44	(0.76–2.71)	0.789	0.92	(0.38–2.24)	0.558
	Moderate-intake group	17	(41.4)	0.90	(0.42–1.95)		1.03	(0.44–2.44)	
	High-intake group	16	(21.2)	1.00			1.00		
Eggs	Low-intake group	32	(36.8)	1.97	(0.87–4.48)	0.060	2.68	(1.07–6.70)	0.037
	Moderate-intake group	12	(43.1)	2.56	(1.14–5.75)		2.59	(1.06–6.33)	
	High-intake group	17	(22.8)	1.00			1.00		

Food group-specific intake (g) is adjusted for energy intake using the residual method; food group-specific intake is classified into three groups (low-intake group, moderate-intake group, high-intake group)

^a^Model I: not adjusted for background factors

^b^Model II: adjusted for age, sleep hours, regular exercise, alcohol consumption, smoking

Multiple logistic regression analysis; Dependent variable: presence or absence of depression; Significance level <0.05 (two-sided test)

In Model II (adjusted for background factors), significantly higher ORs (95% CIs) for depression were found in the following groups compared with the high-intake group: in men, these included the eggs low-intake group (2.59 [1.21–5.54]) and in women, these included the other vegetables low-intake group (2.86 [1.11–7.36]), other vegetables moderate-intake group (2.72 [1.09–6.82]), and eggs moderate-intake group (2.52 [1.08–5.90]). Additionally, a dose-response relationship was observed, where the OR for depression was significantly higher in men when the intake of eggs was low (*p* for trend = 0.024) and in women when the intakes of other vegetables (*p* for trend = 0.011) and eggs (*p* for trend = 0.032) were low.

## Discussion

This study aimed to clarify the relationship between food group-specific intake and depression according to sex among employees. The results showed a dose-response relationship, in which a higher intake of eggs was associated with a lower risk of depression in both men and women. This result is consistent with those of previous studies conducted in rats [30] and among Iranian individuals [31].

Decreased plasma tryptophan concentrations have been reported in patients with depression [45]. Furthermore, individuals with a family history of mental disorders are more likely to feel depressed than are healthy people when provided with an amino acid drink that is low in tryptophan content [46]. Studies have mainly focused on serotonin and serotoninergic neurotransmission when exploring the causes of depression [47]. Serotonin deficiency and depressive symptoms are causally linked. Tryptophan is converted to 5-hydroxytryptophan and subsequently converted to the neurotransmitter serotonin, which may increase the level of serotonin in the brain and improve the depressive symptoms [48]. Eggs are rich in tryptophan; in both men and women, egg intake may have led to a higher tryptophan intake, resulting in an inverse dose-response relationship between egg intake and depression. Although seafood, of which the intake has been associated with depression in previous studies [15, 16, 25, 28], contains large amounts of tryptophan, similar to eggs, no such association was observed in the present study.

With regard to vegetable intake, which has often been associated with depression [20–27], a dose-response relationship was also found in the present study; the intake of other vegetables was inversely correlated with depression among women. Vegetable intake becomes challenging when an individual experiences fatigue [49]; therefore, severe depressive symptoms could be an obstacle to the consumption of vegetables. Furthermore, studies of the intestinal flora of patients with depression have been conducted in recent years [50]. Berk et al. reported that the status of intestinal bacteria differs between healthy individuals and those with depression, with disturbances in the intestinal flora leading to increased intestinal permeability. This increases the entry of toxins and substances, subsequently leading to inflammation and depression [51]. Vegetable consumption may be associated with depression because it improves the intestinal environment and reduces inflammation. Many studies conducted in Europe and the United States have reported an inverse correlation between the Mediterranean diet (that contains a large amount of vegetables) and depression [52, 53]. Additionally, our results are consistent with those of previous studies, which showed that a higher vegetable intake lowers the risk of depression [20–27]. One of the targets of “Health Japan 21 (Second)” is the consumption of at least 350 g of vegetables per day in order to maintain a healthy lifestyle and prevent lifestyle-related diseases [54]. However, the 2019 National Health and Nutrition Survey [55] reported average overall vegetable intakes of 280.5 g—288.3 g in men and 273.6 g in women—with an overall deficit of 70 g. Efforts to increase vegetable intake are necessary not only for preventing lifestyle-related diseases but also for promoting mental health among employees.

Although an association between the Mediterranean diet and depression has been reported [52, 53], and seafood and depression may be associated, no significant association was found in this study. Yonezawa City in Yamagata Prefecture is not adjacent to an ocean; consequently, fish intake is presumed to be low. This may account for an absence of association with depression.

To the best of our knowledge, no other studies involving Japanese participants have reported an association between egg intake and depression. Another strength of this study is the high response rate of 88.6%.

Nevertheless, this study also has certain limitations. It is cross-sectional in nature; therefore, causality cannot be inferred. Matsuda et al. reported a mean CES-D score of 13.2 points in men, with 28.8% having depression [29]. Additionally, the percentage of Japanese individuals with depressive symptoms reported in previous surveys ranges from 27.8% to 37% [10, 17, 56]. These results suggest that the participants in the present study did not have particularly high levels of depression. However, our participants were employees of a specific local government. In addition, coronavirus disease (COVID-19) was prevalent during the study period (October to November 2020), and many reports mention associated changes in mental health [57] and eating habits [58]. Therefore, we cannot exclude the possibility that COVID-19 may also have influenced the results of this study.

## Conclusions

This cross-sectional study found that the odds of depression were higher in both men and women with a low egg intake, and in women with a low vegetable intake. These results suggest that egg and vegetable intake may be associated with depression.

## Data Availability

All data generated or analysed during this study are included in this published article.

## Abbreviations

BDHQ: Brief self-administered diet history questionnaire
BMI: Body mass index
CES-D: Center for Epidemiologic Studies Depression scale
CIs: Confidence intervals
COVID-19: Coronavirus disease
ORs: Odds ratios
